# Interruption of *Onchocerca volvulus* transmission in Northern Venezuela

**DOI:** 10.1186/1756-3305-6-289

**Published:** 2013-10-07

**Authors:** Jacinto Convit, Harland Schuler, Rafael Borges, Vimerca Olivero, Alfredo Domínguez-Vázquez, Hortencia Frontado, María E Grillet

**Affiliations:** 1 Servicio Autónomo Instituto de Biomedicina & Servicio Regionales de Dermatología Sanitaria, Ministerio del Poder Popular para la Salud, Caracas, Venezuela; 2 Onchocerciasis Elimination Program for the Americas, Guatemala City, Guatemala; 3 Instituto de Altos Estudios “Dr. Arnoldo Gabaldón”, Ministerio del Poder Popular para la Salud, Maracay, Venezuela; 4 Laboratorio de Biología de Vectores y Parásitos, Instituto de Zoología y Ecología Tropical, Facultad de Ciencias, Universidad Central de Venezuela, Apartado Postal 47072, Caracas 1041-A, Venezuela

**Keywords:** Onchocerciasis, *Simulium metallicum*, Interruption, Ivermectin, Venezuela

## Abstract

**Background:**

Onchocerciasis is caused by *Onchocerca volvulus* and transmitted by *Simulium* species (black flies). In the Americas, the infection has been previously described in 13 discrete regional foci distributed among six countries (Brazil, Colombia, Ecuador, Guatemala, Mexico and Venezuela) where more than 370,000 people are currently considered at risk. Since 2001, disease control in Venezuela has relied on the mass drug administration to the at-risk communities. This report provides empirical evidence of interruption of *Onchocerca volvulus* transmission by *Simulium metallicum* in 510 endemic communities from two Northern foci of Venezuela, after 10–12 years of 6-monthly Mectizan® (ivermectin) treatment to all the eligible residents.

**Methods:**

In-depth entomologic and epidemiologic surveys were serially conducted from 2001–2012 in selected (sentinel and extra-sentinel) communities from the North-central (NC) and North-east (NE) onchocerciasis foci of Venezuela in order to monitor the impact of ivermectin treatment.

**Results:**

From 2007–2009, entomological indicators in both foci confirmed that 0 out of 112,637 *S. metallicum* females examined by PCR contained L_3_ infection in insect heads. The upper bound of the 95% confidence intervals of the infective rate of the vector reached values below 1% by 2009 (NC) and 2012 (NE). Additionally, after 14 (NC) and 22 (NE) rounds of treatment, the seasonal transmission potential (±UL CIs) of *S. metallicum* was under the critical threshold of 20 L_3_ per person per season. Serological analysis in school children < 15 years-old demonstrated that 0 out of 6,590 individuals were harboring antibodies to Ov-16. Finally, epidemiological surveys made during 2010 (NC) and 2012 (NE) showed no evidence of microfilariae in the skin and eyes of the population.

**Conclusions:**

These results meet the WHO criteria for absence of parasite transmission and disease morbidity in these endemic areas which represent 91% of the population previously at-risk in the country. Consequently, the two Northern foci are currently under post-treatment onchocerciasis surveillance status in Venezuela.

## Background

Onchocerciasis is a chronic infection caused by the filarial worm *Onchocerca volvulus* (Leuckart) and transmitted exclusively to humans through the bites of black fly species of the genus *Simulium* Latreille. Parasite impact on human health is through the clinical repercussions of the infection of the skin and eyes. *Onchocerca volvulus* embryonic forms (microfilariae [mf]) migrate through the skin and cause severe itching, disfiguring disease, and ocular lesions. Visual loss and blindness can be the result of heavy parasite loads in the human host over time.

In the Americas, thirteen onchocerciasis foci have been described in Brazil, Colombia, Ecuador, Guatemala, Mexico and Venezuela, where about 379,234 persons were considered at risk of infection as of 2013 [1]. In this region, several ecological settings are associated with distinct but well-adapted *Onchocerca–Simulium* complexes which show various parasite transmission intensities, degrees of infection severity, clinical manifestations, and epidemiological patterns. In Venezuela, the epidemiology of onchocerciasis is heterogeneous with two contrasting endemic regions. There is a northern endemic area localized in the coastal mountain (“Cordillera de la Costa”) composed of two disease foci that are geographically separated but similar in their epidemiology, namely the North-central [2] and North-east [3] foci. Here, about 108,968 persons from the rural population are at risk [4] and infection is transmitted by *Simulium metallicum* sensu lato Bellardis [5-7]. In contrast, the southern onchocerciasis Amazonian focus is confined to the rainforest of the Upper Orinoco River region, affecting about 10,390 people [4] from the indigenous Yanomami population [8,9]. In this second endemic area, *S. guianense* s.l. Wise, *S. incrustatum* Lutz, and *S. oyapockense* s.l. Floch and Abonnenc are the vectors [10-12].

The main approach taken in the Americas to battle onchocerciasis has been to eliminate the parasite by using mass drug administration, MDA [13,14]. Specifically, the Onchocerciasis Elimination Program for the Americas (OEPA), a regional partnership program founded in 1992, has relied on the mass administration of ivermectin (Mectizan®, Merck & Co Inc) in order to eliminate new ocular morbidity produced by *O. volvulus* and interrupt transmission of the parasite by the year 2015 [1,13]. Ivermectin is a drug that kills the mf in the skin and temporarily inhibits their release by gravid adult worms [15] as well as killing adult worms after several years of mass treatment given at 6-monthly intervals [14,16]. In the Americas, a key and successful strategy has been to employ regular semi-annual and, lately, tri-monthly treatment rounds with coverage (proportion of the population treated) higher than 85% of the eligible population [1,13,17,18]. Indeed, the onchocerciasis elimination program in Venezuela, as those in the other 5 endemic Latin American countries, has relied on this health strategy since 2001.

The present work reports for the first time in Venezuela the interruption of *O. volvulus* transmission in 510 endemic communities localized in the Northern area of the country after 10–12 continuous years of ivermectin treatment. In the Americas, baseline and further clinical, parasitological, ophthalmological and entomological evaluations carried out in selected communities within each regional focus every 4 years have allowed us to monitor the impact of Mectizan® administration on the transmission of *O. volvulus*. We present here the results obtained from several in-depth epidemiological and entomological follow-up studies carried out in the two northern foci from 2001 onwards. This success now allows us to state onchocerciasis transmission is interrupted in this area according to the WHO criteria [19,20].

## Methods

### Study area

**Figure 1:**
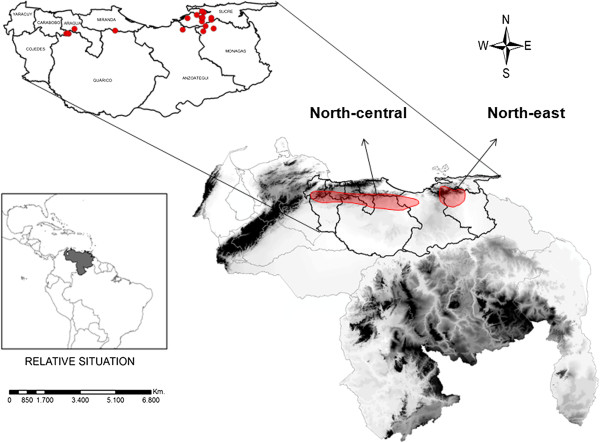
**Map of Venezuela.** Map of Venezuela (including topographic layer) showing the nine administrative boundaries (States) where the onchocerciasis has been endemic in the northern region of the country. Red areas over the map represent the two endemic foci extent. Red points over the enlarged States correspond to those communities (sentinel and extra-sentinel) were regular in-depth epidemiological evaluations (EEPs) were conducted in order to monitor the impact of treatment on parasite transmission in both foci.

The onchocerciasis North-central focus [2] encompasses 6 administrative States (Figure 1) and 45 endemic communities. The population at-risk (14,835 individuals) corresponds to about 12% of the total at-risk population in the country. The North-east focus [3], by contrast, includes 3 administrative States (Figure 1) but 465 endemic communities of about 94,583 inhabitants corresponding to 79% of the total at-risk population in the country. The residents of both endemic areas are mainly part of the rural population dedicated to agricultural activities [7] and the parasite is transmitted by the predominant human-biting black fly *Simulium metallicum* s.l. [5-7]. In this geographical area, the annual mean temperature is about 24°C - 27°C and the total annual rainfall is 1100 mm, with a rainy season from May to October and a dry season from November to April [7]. The main onchocerciasis transmission season occurs at the end of the rainy season and beginning of dry season [21].

### Survey communities: sentinel and extra-sentinel communities

From 1997 to 1999, the Venezuelan National program, coordinated by the local health authorities (Regional Dermatology Services) and the Biomedicine Institute compiled the complete epidemiological history and endemic status of the disease in the northern area according to previously published reports from the Venezuelan Minister of Health. This pre-ivermectin information allowed the basal epidemiological stratification of all the communities in both foci (North-central and North-east).

In the North-central focus (NC), a total of 45 communities were classified - 42 hypoendemic (prevalence of mf infection < 20%), two mesoendemic (prevalence ≥ 20% but lower than 60%) and one hyperendemic (prevalence ≥ 60%). Here, ocular pathology (about 37.9% and 31% prevalence of microfilariae in the cornea [MFC] and the anterior chamber of the eye [MFAC], respectively) was the major clinical manifestation attributable to onchocerciasis according to previous records. By contrast, skin disease (about 1.2 community microfilariae load [CMFL]) and pruritus were minor manifestations, whereas parasite nodules and blindness were absent. From the 45 communities, a total of 1 sentinel and several extra-sentinel communities were selected as those communities where regular in-depth epidemiological evaluations (EEPs) would be conducted in order to monitor the impact of treatment on parasite transmission (Figure 1). Santa Rosa del Sur (825 m.a.s.l.), having a current population of 129 inhabitants, was chosen as the sentinel community (70% of mf basal prevalence). La Llanada (713 m; 119 inhabitants; 48.2% of basal prevalence), Virgen Pura (849 m; 115 inhabitants; 23.8% of basal prevalence) and El Chino (854 m; 137 inhabitants; 12.5% of basal prevalence) were selected as the extra-sentinel communities. A fourth extra-sentinel hypo-endemic community was used (Buena Vista: 547 m; 174 inhabitants) for entomological evaluations in order to increase the statistical certainty (higher number of collected flies) of interruption of parasite transmission.

In the North-east focus, a total of 233 hypoendemic, 197 mesoendemic and 35 hyperendemic communities were identified prior to the beginning of ivermectin treatment. Here, the basal ocular pathology was ≈ 27.8% (MFC) and 25.3% (MFAC), whereas skin disease (CMFL ~ 0.58) and pruritus were minor manifestations. Parasite nodules and blindness were absent. From the 465 communities, a total of 5 sentinels and 8 extra-sentinels communities were selected (Figure 1). La Carapa (697 m; 202 inhabitants; 73.3% of basal prevalence), Voladero (628 m; 202 inhabitants; 60% of basal prevalence), Santa Marta (118 m; 184 inhabitants; 62.5% of basal prevalence), Caituco (98 inhabitants; 72.7% of basal prevalence), and La Cuesta (261 m; 218 inhabitants; 68.8% of basal prevalence) were the sentinel communities. The extra-sentinel communities were: Manapire Abajo (508 m; 315 inhabitants; 76.7% of basal prevalence), Guayabal (468 m; 206 inhabitants; 65.5% of basal prevalence), El Filudo (570 m; 99 inhabitants; 28% of basal prevalence), El Naranjal (436 m; 86 inhabitants; 75% of basal prevalence), El Piñal (71.4% of basal prevalence), Sabaneta (422 m; 160 inhabitants; 87.5% of basal prevalence), Jenjibral (414 m; 36 inhabitants; 44.4% of basal prevalence), and Apamatal (453 m; 30 inhabitants; 55.6% of basal prevalence).

**Figure 2:**
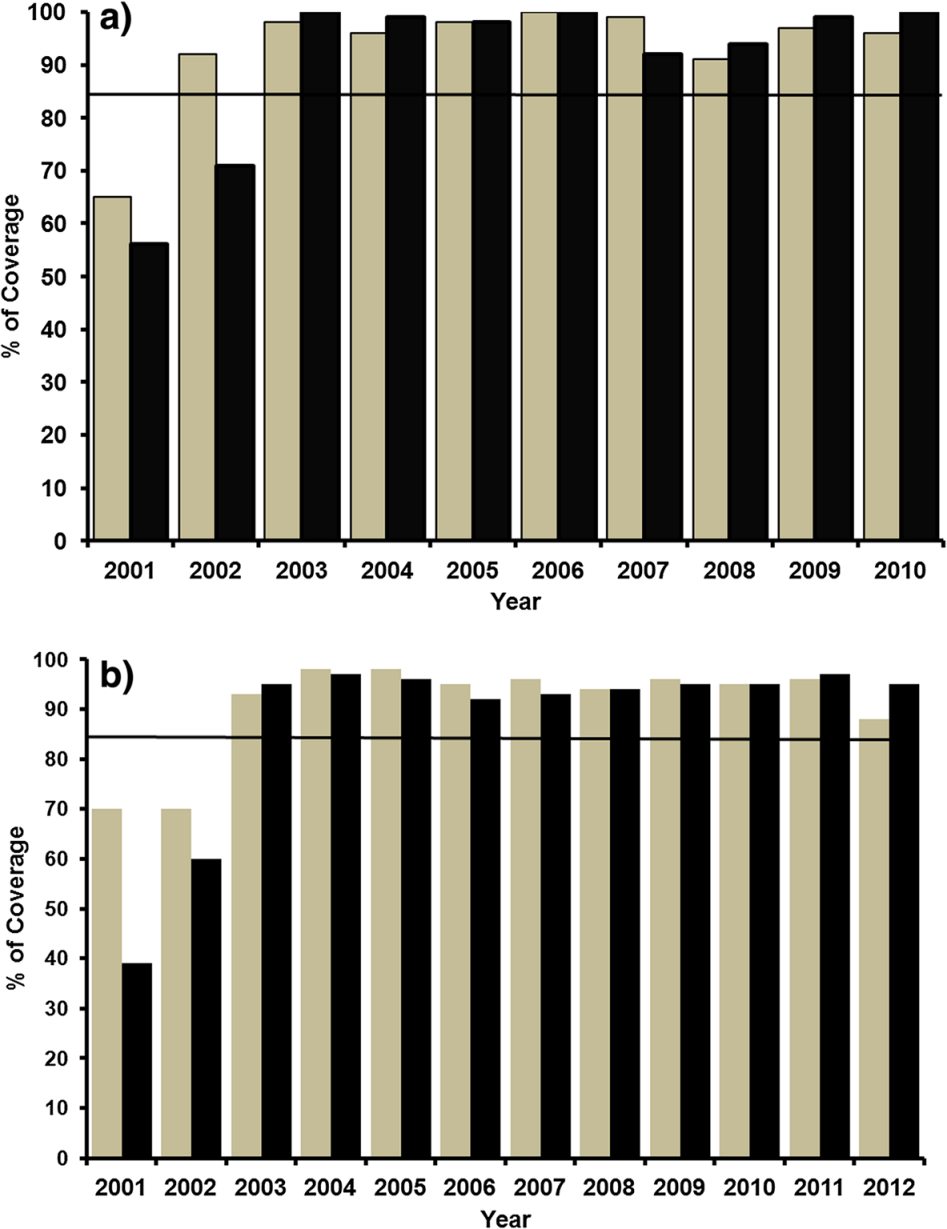
**Coverage rate with ivermectin, northern Venezuela.** Coverage rate (percent) with ivermectin of the eligible population of the North-central **(a)** and North-east **(b)** foci, Venezuela, 2001–2012. The line at 85% indicates the coverage needed in a sustained way to interrupt transmission.

In-depth epidemiological evaluations carried out from 2001 onwards allowed us to update the endemic status of both foci. Most of the above communities showed lower disease prevalence than previously reported, with most of the communities lying in hypoendemic and mesoendemic disease levels. The onchocerciasis elimination program in Venezuela (OEPV) started during 2001 the 6 monthly regular mass treatments with Mectizan® to every eligible resident in all the affected communities from the Northern area of Venezuela (Figure 2). The 85% goal of coverage treatment was reached two (North-central) and three (North-east) years later and that level remained until 2010 (Figure 2a) and 2012 (Figure 2b), when each focus had reached 20 (North-central) and 24 (North-east) regular treatment rounds, respectively.

### WHO criteria to onchocerciasis elimination

In 2001 WHO published the document “Certification of elimination of human onchocerciasis: criteria and procedures,” which established the different phases to be followed by a country to achieve certification of elimination of onchocerciasis [19]. Each phase is associated with an aspect of parasite transmission resulting in four phases. More recently, OEPA, through its program coordinating committee (PCC), published a field document that has as its focus the 3-year period defined by post-treatment surveillance (PTS) and describes the activities that distinguish and bridge phase *transmission interrupted* and phase *transmission eliminated*[20]. Thus, the following phases have been recognized and pursued by each country in the Americas to achieve the goal of parasite and disease elimination [19,20]. *Stage 1* is the phase of *ongoing* transmission characterized by the presence of *O. volvulus* infective larvae (L_3_ stage) in the vector population (insect heads) and parasite mf in skin, parasite nodules and serology in the population and especially in children < 5 year-old. *Stage 2* is when most of the above transmission indicators start to show negative results, consequently, the status of the focus changes to *suppressed* transmission. In *Stage 3,* transmission is regarded as *interrupted* when the focus (in overall) has reached specific epidemiological indicators such as: i) prevalence of < 1% of *O. volvulus* mf in the cornea and/or anterior chamber of the eye, ii) an infectivity rate (L_3_ infection in heads) by PCR of < 1/1000 (0.1%) in parous flies or <1/2000 (0.05%) in all flies, assuming a 50% parous rate, iii) an annual transmission potential (ATP) or seasonal transmission potential (STP) under 20 L_3_s per time period, and iv) a reduction of new infections to an incidence rate of less than one new case per 1,000 individuals (<0.1%) defined as lack of specific Ov-16 antibodies to *O. volvulus* in school children. In this phase, suspension of treatment is recommended and a 3-year period post-treatment surveillance is initiated in the focus. *Stage 4* occurs after the 3 years of post-treatment surveillance, when the surveillance parameters have been carried out and all results confirm no recrudescence of disease. Consequently, onchocerciasis is declared *eliminated* by WHO after the country requests evaluation [19,20]. Requests for certification of elimination are not made by focus, but by country. However, the report by focus of the empirical evidences about the local interruption (*Stage* 3) and/or elimination (*Stage* 4) of disease transmission will contribute, progressively, to support the final request for certification of each country.

### Entomologic indicators

Based on the previous guidelines, in the sentinel and extra-sentinel communities, and during several (e.g., three to five) consecutive collection days, host-seeking *S. metallicum* females were collected throughout the transmission season (September to March) by using standardized procedures [11,20]. In the North-central focus, entomological collections were performed from September 2007 to March 2008 in the sentinel community, and from September to November 2009 in the extra-sentinel communities. For the North-east focus, black fly collections were performed from September 2007 to March 2008, and during 2011 in the 5 sentinel communities. Similar entomological catches were carried out during 2010 (September to November) and 2012 (September to November) in 5 extra-sentinel communities. All the simuliid females that landed on two human attractants from each community were caught with manual aspirators by a team of two collectors and two attractants during the first 50 minutes of each hour, beginning at 09:00 hours and ending at 17:50 hours, with one stop hour (12:00 – 13:00). This period coincided with the highest number of parous females of *S. metallicum* (unpublished results). This amounted to 8 collection periods per catching day. Females were collected before procuring a blood meal. Attractants received Mectizan® 1 week before beginning the collection process. Whenever possible, the collection team was the same throughout the surveys to minimize variations resulting from individual differences in catching ability. In the field, all hourly-caught flies were anesthetized with chloroform vapour, identified to species, and counted by the hour, day, date, and community. The number of collection days depended on the biting density for each community and followed criteria to calculate a more precise transmission potential (TP) index (see below). Polymerase chain reaction (PCR) using *O. volvulus* -specific DNA probes (see below) is generally applied to examine pools of flies in the OEPA region [19,20]. Therefore, by each community, flies were combined into pools containing a maximum of 50 individuals per pool and the heads and bodies tested separated for *O. volvulus* by using a PCR assay specific for this parasite species [22]. Details of protocols for genomic DNA purification and parasite detection have been published elsewhere [22,23]. Body pools were analyzed first; if any of the body pools were positive, the body testing was repeated. If the positive body pool was confirmed then body pool testing was suspended and all of the head pools were then analyzed. Positives were confirmed by a second PCR as part of a process to standardize this procedure from the University of South Florida [20]. Since the female parity status (proportion of females that have already laid a batch of eggs) is usually hard to determine in black fly catches in the field, the OEPA entomological criterion for interruption of transmission or transmission threshold is less than one infective fly per 2,000 flies tested (assuming 50% of these are parous flies). To reach this standard, it has been determined that the minimum sample size required to exclude a prevalence of infective flies of 0.05% in all flies at a 95% CI, given that no infective fly is found, is roughly 6,000 flies by focus [20]. In addition to these criteria, during 2006, an OEPA-convened meeting of entomologists recommended the use of ATP or STP to further assess the status of onchocerciasis transmission in the Americas, because both of these measurements take into account the biting rate and the prevalence of infective flies [20,24]. All entomologists at that meeting agreed that an ATP < 20 L_3_s/person/year represented interruption of parasite transmission, based on previous theoretical [25] and field [26] estimates carried out in the region.

Regarding the northern endemic area of Venezuela, pre-treatment entomological surveys carried out during 1998–1999 in some of the sentinel communities found that the *S. metallicum* infectivity rates (proportion of flies with infective-stage *O. volvulus* larvae per 2000 flies examined) varied from 0.0003 (95% CIs: 0.0001-0.006) to 0.0009 (95% CIs: 0.0005-0.0014) in the North-east focus; whereas the infectivity rate showed values of 0.0005 (95% CIs: 0.0003-0.0008) in the only sentinel locality of the North-central focus [27].

### Serologic indicators

Here, the objective was to measure the prevalence of IgG4 antibodies to Ov-16, a recombinant pre-patent antigen of *O. volvulus*[28-30]. The OV-16 testing in ELISA is currently being used in sero-surveys in areas where transmission is thought to be interrupted in the Americas region [20]. For that, three serological surveys were carried out in the eligible young population (< 15 years old) from the 45 endemic communities of the North-central focus during 2008, 2009 and 2010. Likewise, three serological surveys (children < 5 years old) were performed during 2006, 2009 and 2012 from the 465 endemic communities of the North-east focus. The sample size required to calculate a one-sided 95% confidence interval (CI) for a point prevalence that excludes 0.1% has been determined to be around 3,000 children or all the children of eligible age in the endemic area. The serological protocol was as follows [30]. Sterile procedures were used to prick the fingers of all participants and four to six drops of blood (80–120 μL) were absorbed onto Whatman No 2 filter paper. The filter paper blood samples were dried, separated by sheets of paper, and then bundled and stored in sealed plastic bags in a cooler until they were returned to the laboratory where they were stored at -20°C. Two 6 mm punches of blood-saturated filter paper were placed in a phosphate-buffered saline (Tween 0.05% and bovine serum albumin 5%) and eluted overnight at 4°C. The elution was then run in duplicate in a standard ELISA to detect IgG4 antibodies against the Ov-16 recombinant antigen [30].

### Parasitological and ophthalmologic indicators

In the North-central focus, a total of 4 parasitological (2001, 2005, 2008, 2010) and 2 ophthalmologic (2008, 2010) surveys were carried out in the sentinel and extra-sentinel communities. Alternatively, 4 parasitological (2001, 2006, 2009, 2012) and 4 ophthalmologic (2001, 2006, 2009, 2012) surveys were performed in the North-east focus.

The skin snip biopsy was the standard method to determine the prevalence of microfilariae in the skin as well as intensity of *O. volvulus* infection in the studied population. Two simultaneous skin biopsies were taken from each patient > 1 year-old from each community by using a Holth sclerocorneal punch, one from the left supra-scapular region and the right supra-iliac region, followed by incubation of the snips for 24 h in buffered saline solution and counting of the emerging microfilariae under a microscope. The prevalence of microfilariae in the cornea (MFC) and/or anterior chamber of the eye (MFAC) was determined by an ophthalmologist experienced in onchocerciasis ocular evaluations.

### Ethical approval

The entomologic and serologic studies received the ethical approval of the Ethic Committee of the Biomedicine Institute (Venezuelan Ministry of Health). All the participants signed an informed consent form before undergoing any examination or testing.

### Data analysis

The infectivity rate in the community and the associated 95% CIs were expressed as the number of positive flies per 2,000 flies examined [22]. The geometric mean number of vectors caught per hour is calculated as [exp (∑log (χ+1) / η) -1] / 0.833, where *χ* + 1 is the number of flies caught in a 50-minute collection period plus 1 (to avoid log [0]), η is the number of collections periods, and 0.833 is the conversion factor to convert a 50-minute collection period into 1 hour [20]. This geometric mean hourly landing rate (which approximates to the biting rate) was calculated for the vector over the capture period. The total biting density for the collection period (called the seasonal biting density, SBD) was calculated as the geometric mean hourly biting rate multiplied by 10 potential hours of biting per day and the number of days in the season. Seasonal transmission potentials (STP) for each sentinel and extra-sentinel village was calculated as the product of the SBD, the proportion of flies with infective-stage *O. volvulus* larvae, and the mean number of infective larvae per infective fly (assumed to be one in an area of low transmission). This entomological indicator can be defined as the number of L_3_s that a person would potentially receive if the individual were maximally exposed to black fly bites during the whole transmission season [31]. The PoolScreen® software program (Version 2.0; University of Alabama, Birmingham, AL) was used to estimate the infectivity rate in the community, that is, the proportion of positive head pools in the PCR assay. This software employs a statistical model to calculate the probability of infection of an individual black fly from the number of positive pools and the size of the pools is used to calculate the proportion of infective flies with 95% CI computed using the Bayesian method [22,32].

## Results

### North-central focus

Results of the entomological evaluations carried out from 2007 to 2009 are presented in Table 1, from which it can be seen that a total of 24,038 *S. metallicum* females were collected in sentinel and extra-sentinel communities (Table 1). *Simulium metallicum* reached a SBR of 99,278 bites/person-season in Santa Rosa del Sur, whilst it was biting at a much lower rate (95%-UL: 24,184 bites/person-season) in the other communities. All the collected flies were examined by PCR in 248 and 269 pools, respectively. Black fly body pools were negative for *O. volvulus* DNA; therefore, insect head pools were not screened. Upper confidence interval limits of the prevalence of infective flies in all the communities were under the critical threshold of 1/2,000 (Table 1). Upper limits of the STPs in all the communities ranged from 2.9 to 19.9 L_3_ per person per season. Table 2 shows the results of the serological studies which revealed that no Ov-16 IgG4 antibodies were occurring in the 2,089 children < 15 years old examined in the studied communities after 8–10 years post-MDA. Finally, the results of the epidemiological surveys are shown in Table 3. The prevalence of *O. volvulus* mf in the sentinel community depicted a significant decline from a prevalence of 2% of mf in skin and a geometric mean of 0.1 mf per skin snip in the community (CMFL) during 2001 to zero levels from 2005 onwards. These figures have maintained that level until 2010 where the focus had reached 20 treatment rounds (Figure 2a). No MFC or MFAC were found among the inhabitants during 2010 (Table 3).

**Table 1:** **Seasonal biting density (SBD), prevalence of infective flies, and seasonal transmission potential (STP) of *S. metallicum *(2007–2009) in the sentinel and extra-sentinels communities of the North-central onchocerciasis focus, Venezuela**

**Community**	No. of flies collected^*^	**Seasonal biting density**^**γ **^**(CI)**^**ψ**^	**Prevalence of infective flies**^**δ **^**(CI)**^**ψ**^	**Seasonal transmission potential**^**θ **^**(CI)**^**ψ**^
Santa Rosa del Sur (Sentinel)	11,370	99,278	0 (0–0.4)	0 (0–19.9)
		(87,847 – 112,167)		
Extra-sentinel Communities^σ^	12,668	19,554	0 (0–0.3)	0 (0–2.9)
		(15,771 – 24,184)		

*Collection period (season): Sentinel (September 2007-March 2008) and other (November to December 2009) communities.

^ψ^The associated 95% Confidence Intervals (CIs) surrounding the point estimate.

^γ^SBD: Mean number (geometric mean) of *S. metallicum* bites per person per season.

^δ^Number of positive (heads) for *O. volvulus* L_3_ per 2,000 flies examined.

^θ^STP: SBD x prevalence of infective flies x mean number of L_3_ per infective fly.

^σ^La llanada, El Chino and Buena Vista.

**Table 2:** Prevalence of IgG4 antibodies to Ov-16 in children (< 15 years old) from the 45 endemic communities of the 6 administrative States of the North-central focus of onchocerciasis, Venezuela

**State**	IgG4 prevalence (positive / no.examined)*
Aragua	0 / 612
Carabobo	0 / 192
Cojedes	0 / 58
Guarico	0 / 476
Miranda	0 / 478
Yaracuy	0 / 273
**Focus**	**0 / 2,089**

* Surveys carried out from 2008 to 2010.

**Table 3:** ***Onchocerca volvulus *infection in the human population (sentinel community of Santa Rosa del Sur), North-central focus, Venezuela**

**Survey period**	**Prevalence of skin mf (%)**	**Community Microfilariae Load (CMFL)**	**Prevalence of MFC (%)**	**Prevalence of MFAC (%)**
2001	2.0	0.01	0.0	0.0
2005	0.0	0.0	1.7	0.0
2008	0.0	0.0	1.8	0.0
2010	0.0	0.0	0.0	0.0

### North-east focus

A total of 88,599 *S. metallicum* females were collected in this focus (Tables 4 and 5) from 2007 to 2012. The biting rate of this species ranged from 55,884 bites/person/season in the community of Caituco to 2,907 bites/person/season in the Voladero community (Table 4). A total of 700 bodies and 2,089 heads pools of *S. metallicum* were examined by PCR, showing a prevalence of 0.06 (43 positive bodies/700) infected flies by 2,000 and 0 (0 positive heads/2089) infective flies by 2,000 during the whole evaluation period (Tables 4 and 5). Regarding the upper confidence interval limits of the prevalence of infective flies, most of the figures by community and by focus were under the critical threshold of 1/2,000, except for 5 sentinel communities (Tables 4 and 5). However, the upper limits of the STPs were below the threshold of 20 L_3_ per person per season, particularly in the Caituco community (Table 4), from 2011 onwards. In Table 6 it can be seen that no Ov-16 IgG4 antibodies were detected in the 3,994 children < 5 years old examined during 2012 from 132 communities within the focus, despite some positive samples were previously found during 2006 (2/106 children) and 2009 (1/289 children). Finally, the epidemiological surveys showed that the prevalence of *O. volvulus* mf in the focus (Tables 7 and 8) diminished from a specific prevalence of 33.3% of mf in skin and a geometric mean of 0.21 mf per skin snip in the El Piñal community (CMFL), as an example, to 0 levels during 2012, after 24 rounds of treatment (Table 8). A similar decline was observed with the MFC and MFAC values in this community by 2012. Three communities (La Carapa, Guayabal and El Filudo) were the exception within the focus, not showing mf prevalence < 1% in the skin (Guayabal) or eyes (La Carapa and El Filudo) during the 2012 survey (Tables 7 and 8). However, when we considered the figures for the whole focus, either the prevalence of mf in the skin (0.3%; CMFL = 0.001) or the prevalence in the eyes (MFC = 0.8%; MFAC = 0.2%) were below the critical threshold of *O. volvulus* transmission by 2012.

**Table 4:** **Seasonal biting density (SBD), prevalence of infective flies (PIF), and seasonal transmission potential (STP) estimates of *S. metallicum *(2007–2012) in the sentinel communities of the north-east onchocerciasis focus, Venezuela**

**Community**	**Flies collected**	**SBD**^**γ **^**(CI)**^**ψ**^	**PIF**^**δ **^**(CI)**^**ψ**^	**STP**^**θ **^**(CI)**^**ψ**^
La Carapa (2007–2008)	12,014	41,764	0 (0–0.3)	0 (0–6.3)
Voladero (2007–2008)	6,192	13,538	0 (0–0.6)	0 (0–4.1)
Santa Marta (2007–2008)	6,201	9,826	0 (0–0.6)	0 (0–2.9)
Caituco (2007–2008)	5,855	55,884	0 (0–0.7)	0 (0–19.6)
**Focus (2007–2008)**	**30,262**	**17,757**	**0**	**0**
		**(16,362 –19,257)**	**(0–0.1)**	**(0–0.9)**
La Carapa (2011)	5,642	18,528	0 (0–0.7)	0 (0–6.5)
Voladero (2011)	1,241	2,907	0 (0–3.1)	0 (0–4.5)
Santa Marta (2011)	2,423	12,799	0 (0–1.6)	0 (0–10.2)
Caituco (2011)	4,155	15,511	0 (0–0.9)	0 (0–7.0)
La Cuesta (2011)	3,255	5,617	0 (0–1.2)	0 (0–3.4)
**Focus (2011)**	**16,712**	**6,199**	**0**	**0**
		**(5,717 – 6,719)**	**(0–0.2)**	**(0–0.6)**
Caituco (2012)	3,923	13,034 (11,652 – 14,569)	0 (0–1.0)	0 (0–3.4)

^ψ^The associated 95% Confidence Intervals (CIs) surrounding the point estimate.

^γ^SBD: Mean number (geometric mean) of *S. metallicum* bites per person per season.

^δ^Number of positive (heads) for *O. volvulus* L_3_ per 2 000 flies examined.

^θ^STP: SBD x prevalence of infective flies x mean number of L_3_ per infective fly.

**Table 5:** **Seasonal biting density (SBD), prevalence of infective flies (PIF), and seasonal transmission potential (STP) estimates of *S. metallicum *in the extra-sentinels communities of the North-east onchocerciasis focus, Venezuela, during 2010 and 2012**

**Community**	**Flies collected**	**SBD**^**γ **^**(CI)**^**ψ**^	**PIF**^**δ **^**(CI)**^**ψ**^	**STP**^**θ **^**(CI)**^**ψ**^
Manapire Abajo (2010)	4,208	18,856	0 (1–0.9)	0 (1–8.5)
Guayabal (2010)	9,491	37,007	0 (0–0.4)	0 (0–7.4)
Sabaneta (2010)	4,135	32,764	0 (0–0.2)	0 (0–3.3)
Naranjal (2010)	7,992	31,056	0 (0–0.5)	0 (0–7.8)
El Filudo (2010)	5,144	21,084	0 (0–0.7)	0 (0–7.4)
**Focus (2010)**	**30,970**	**39,155**	**0**	**0**
		**(36,703 – 41,763)**	**(0–0.1)**	**(0–2.0)**
Guayabal (2012)	2,411	10,281 (9,612–11,700)	0 (0–1.6)	0 (0–8.2)
Naranjal (2012)	4,321	10,453 (9,256–11,786)	0 (0–0.9)	0 (0–4.7)

^ψ^The associated 95% Confidence Intervals (CIs) surrounding the point estimate.

^γ^SBD: Mean number (geometric mean) of *S. metallicum* bites per person per season.

^δ^Number of positive (heads) for *O. volvulus* L_3_ per 2 000 flies examined.

^θ^STP: SBD x prevalence of infective flies x mean number of L_3_ per infective fly.

**Table 6:** Prevalence of IgG4 antibodies to Ov-16 in children (up to 5 years old) from 132 endemic communities of the 3 administrative States of the North-east focus of onchocerciasis, Venezuela

**State**	IgG4 prevalence (positive / no.examined)*
Sucre	0 / 2,069
Monagas	0 / 899
Anzoategui	0 / 1,026
**Focus**	0 (0 – 0.09)^γ^ / 3,994

* Surveys carried out during 2012.

^γ^Associated 95% Confidence Intervals (CIs).

**Table 7:** ***Onchocerca volvulus *infection in the sentinel communities, North-east focus, Venezuela**

**Community**	**Survey period**	**Prevalence of skin mf (%)**	**Community Microfilariae Load (CMFL)**	**Prevalence of MFC (%)**	**Prevalence of MFAC (%)**
La Carapa	2001	3.0	0.03	2.3	2.3
	2005	3.6	0.03	0.0	0.0
	2009	0.7	0.004	0.0	0.8
	2012	0.0	0.0	1.0	0.0
Voladero	2001	3.0	0.02	-	-
	2005	4.0	0.02	-	-
	2009	2.5	0.01	-	-
	2012	0.0	0.00	-	-
Santa Marta	2001	3.0	0.03	-	-
	2005	4.2	0.07	-	-
	2009	0.7	0.01	-	-
	2012	0.0	0.00	-	-
Caituco	2001	15.0	0.12	-	-
	2005	6.8	0.05	-	-
	2009	4.8	0.04	-	-
	2012	0.0	0.00	-	-
La Cuesta	2001	3.0	0.01	-	-
	2005	1.7	0.01	-	-
	2009	0.7	0.01	-	-
	2012	0.0	0.01	-	-

**Table 8:** ***Onchocerca volvulus *infection in extra-sentinel communities, North-east focus, Venezuela**

**Community**	**Survey period**	**Prevalence of skin mf (%)**	**Community Microfilariae Load (CMFL)**	**Prevalence of MFC (%)**	**Prevalence of MFAC (%)**
Manapire Abajo	2006	5.1	0.04	0.7	0.7
	2009	0.9	0.01	0.0	0.0
	2012	0.0	0.0	0.5	0.0
Guayabal	2006	16.7	0.19	1.3	6.5
	2009	12.8	0.16	0.9	1.8
	2012	2.0	0.01	0.8	0.0
Filudo	2006	14.9	0.28	0.0	6.5
	2009	13.9	0.16	0.0	1.7
	2012	0.0	0.0	1.8	1.8
El Piñal	2006	33.3	0.21	0.0	16.7
	2009	7.7	0.05	0.0	0.0
	2012	0.0	0.0	0.0	0.0
Jenjibral	2006	5.3	0.07	-	-
	2009	0.0	0.0	-	-
	2012	0.0	0.0	-	-
Apamatal	2006	8.0	0.03	-	-
	2009	0.0	0.0	-	-
	2012	0.0	0.0	-	-

## Discussion

Interruption of *O. volvulus* transmission is defined as the reduction of parasite infection to such levels (below specific parasite density breakpoints) that local transmission can no longer sustain the population [20,31]. This work has reported the entomological and epidemiological evidences that this disease stage has been reached in the two Northern foci of Venezuela after 10 (North-central focus) and 12 (North-east focus) continuous years of Mectizan® treatment (Figure 2) administered twice a year to 510 endemic communities in the region.

The first unequivocal signal of current *O. volvulus* transmission in an endemic area is the presence of infective larvae in the head of the vector fly. Here, entomological evaluations carried out in the sentinel and extra-sentinel communities of both foci showed the absence of infective-stage (L_3_) larvae of *O. volvulus* in the *S. metallicum* biting populations suggesting that the parasite-vector contact has not been taking place in this endemic area since 2007; this is, after 6 years of consecutive human population twice-yearly treatment with Mectizan® (Tables 1, 4, and 5). Pre-treatment entomological surveys carried out in the sentinel communities of the northern endemic area of Venezuela had found that the *S. metallicum* infectivity rates varied from 0.0003 (La Carapa) to 0.0009 (La Cuesta) in the North-east focus, whereas the infectivity rate showed values of 0.0005 in the sentinel locality of the North-central focus [27]. In our surveys, vector infectivity rates and their corresponding UL 95% CIs were below 1/2,000 of the transmission critical threshold [19,20] in both foci, except for some communities in the North-east focus. However, all the seasonal potential transmission values and their corresponding UL 95% CIs were below 20 L_3_ per person per season in both foci, including the above mentioned communities (Tables 1, 4 and 5).

Another strong indicator demonstrating the interruption of transmission was the absence of antibodies to the antigen Ov-16 due to recent exposure to *O. volvulus* in children up to 5 years of age that had not received treatment [19]. A 5 year cumulative incidence rate with less than 1 new case per 1,000 susceptible children is acceptable provided that the appropriate population size is available. In the North-central focus (Table 2), it was difficult to find grouped preschool children under the age of 5 years accessible for sampling because some parents were reluctant to let very young children submit to bloodletting. Consequently, we tested 2,089 children up to 15 years old throughout the 45 endemic communities which still fulfilled the WHO criteria [20]. By contrast, a total of 3,994 children up to 5 years old were tested by the presence of antibodies to the antigen Ov-16 during 2012 in the North-east focus (Table 6). In both epidemiological settings, we did not detect specific antibodies to *O. volvulus,* these results imply that no new infections are occurring in the area. The support for transmission interruption is even stronger if we use children older than 5 years old since acquisition of new *O. volvulus* infection rises fastest between 5 and 20 years of age [20].

Prevalence of < 1% of *O. volvulus* mf in the cornea and anterior chamber of the eye as well as < 1% of *O. volvulus* mf in the skin were the last WHO criterion satisfied by our surveys to confirm the interruption of the parasite in the 6 sentinel communities of both foci. Overall, all the examined population had < 1 mf for the skin and eyes by the 2010 (North-central focus) and 2012 (North-east focus) years, respectively, except for 3 communities in the North-east focus. These last results were accounted for by 3 identified persons (one each community) who had left the communities and escaped drug treatment during the last years of MDA; thus, particular control measures were applied to these persons at the end of 2012. Currently, our findings strongly suggest that neither ocular nor skin disease is attributable to *O. volvulus* infection in the Northern area of Venezuela.

Since the intensity of transmission and prevalence of infection in both foci have fallen below accepted threshold values [19,20], this is the first evidence that interruption of onchocerciasis has been accomplished in the North endemic area of Venezuela. This means that around 91% of the population previously at risk in the country are no longer affected. A total of 20–24 semi-annual regular treatment rounds with high coverage (> 85% of the eligible population) have been very important and effective to reach the goal of interruption of transmission (Figure 2). Since the human population represents the only reservoir of human onchocerciasis, this has been a critical biological factor when considering control strategies in the Americas [14]. Mectizan® reduces ≥ 95% of the skin microfilarial load up to 2 months of treatment [15,16], consequently, decreasing or blocking infection of the black flies [31]. It also has an embryostatic effect on adult female worms (approximately 70%), temporarily blocking the release of mf [15] as well as significantly reducing insemination of females [16]. Consequently, local elimination of the parasite can be feasible within this epidemiological period due either to: i) mono-infections (a single adult male or female worm) ii) female worms not being fertilised, thus, not producing microfilariae, or iii) very few microfilariae being produced and ingested by vector flies in numbers insufficient to maintain viable annual transmission levels [31]. Indeed, simulation models (EuSIMON) [33] developed for both Northern foci after the 10–12 years of ivermectin treatment predict that local transmission cannot maintain itself and the adult infection will die out over time. Reintroduction of the parasite from other areas is not likely since the disease has been confined to very small and relatively isolated areas in the northern mountainous region of the country (Figure 1). On the other hand, onchocerciasis models and theory predict that risk of recrudescence will depend on the pre-control endemicity level and the vector species efficiency as indicators of the local potential of parasite transmission [25]. The pre-control endemicity of both foci (the prevalence and intensity of infection in the human population) was relatively low compared to the southern focus of the country [9]. As an example, in the northern onchocerciasis foci, the community microfilarial load (CMFL) before mass drug administration never reached levels above 5 mf per mg of skin in contrast to the southern focus figures [9]. In addition, *S. metallicum* has a relatively low vectorial competence [7] compared to other Venezuelan and American vector black fly species [12,31], regardless of its moderate to high biting density (mean monthly biting rate ranging from 14, 284 to 34, 428 bites per person per month; unpublished results).

WHO certification guidelines for onchocerciasis elimination recommend that, in areas where transmission has been interrupted and MDA has been discontinued, post-treatment surveillance (PTS) should be implemented for 3 years [19,20]. If no recrudescence of infection is detected during this time, then *O. volvulus* can be declared to have been eliminated and the resident population no longer at risk. Based on these criteria, the Program Coordinating Committee (PCC) of OEPA recommended to health authorities from the country to stop MDA by 2011 (North-central focus) and 2013 (North-east focus), respectively. Following that advice, post-treatment surveillance is currently ongoing in this northern endemic area of Venezuela.

Our results contribute to the success of the OEPA strategy [1,4,13,14,34] since the active programs of each of the other five countries in Latin America have also made significant progress regarding onchocerciasis transmission status [4,35-38]. Currently, no new cases of disease blindness have been reported in the region and the ocular morbidity has been eliminated from eleven of the 13 foci. Parasite transmission has been interrupted in eleven (about 96% of the total population at risk and representing four of the six countries where the disease was formerly endemic) with elimination from 7 (33% of the total at risk) foci. In 2013, onchocerciasis was declared eliminated, for the first time, in Colombia, one of the 6 endemic countries, whereas by 2014, Ecuador could become the second Latin American country in reaching that goal [1]. Indeed, the prospect of eliminating onchocerciasis from Africa by mass ivermectin alone has been revived following recent studies in Mali, Nigeria and Senegal that have indicated that annual (or biannual) ivermectin distribution may lead to local elimination of onchocerciasis in certain African foci [39-41].

## Conclusions

We have reported evidences of the local interruption of *O. volvulus* transmission by *Simulium metallicum* in 510 endemic communities localized in Northern Venezuela after 10–12 continuous years of 6-monthly ivermectin treatment. The absence of parasite infective larvae in the black fly vectors, the lack of embryonic parasite forms in the skin and eyes, and the reduction of exposure to and new infections with *O. volvulus* have been the WHO criteria followed to certify interruption of parasite transmission. Together, the results presented here also indicate that onchocerciasis infection no longer poses a significant public health risk in this northern endemic area in Venezuela. Currently, treatment with Mectizan® has been stopped and post-treatment surveillance is ongoing in the area.

The remaining transmission zone in Venezuela, the southern onchocerciasis Amazonian focus [9], is the only active focus in the Americas shared by Brazil [4]. This endemic Yanomami area extends through remote and densely forested zones and is populated by the Yanomami people, a migratory indigenous group that routinely moves across the border at will [4,9]. In 2006, the 85% goal of coverage treatment with ivermectin was reached by the Venezuelan focus and afterward, in-depth epidemiological evaluations have shown that infection intensity and infective flies have decreased by 99%, and eye disease (due to corneal lesions induced by microfilarial death) by 96% in some sentinel communities [9]. Since 2009, the Venezuelan program has introduced the 3-monthly mass treatment in an attempt to interrupt transmission in this geographical area by 2015 [1,4]. The seminomadic characteristics of the human population, the extent and remoteness of the Yanomami area and the continuing discovery of new endemic communities on the Venezuelan side are the biggest challenges to face in promptly interrupting disease transmission throughout the Amazonian region; however, new approaches and strategies are being considered to address the issue at country and international levels.

## Competing interests

The authors declare that they all have no competing interests.

## Authors’ contributions

HS, VO, MEG, AD and HF designed and performed the data collection and studies. AD and MEG analyzed the data. MEG wrote the paper. All authors read and approved the final manuscript.
